# Stability of beating frequency in cardiac myocytes by their community effect measured by agarose microchamber chip

**DOI:** 10.1186/1477-3155-3-4

**Published:** 2005-05-31

**Authors:** Kensuke Kojima, Tomoyuki Kaneko, Kenji Yasuda

**Affiliations:** 1 Department of Life Sciences, Graduate school of Arts and Sciences, University of Tokyo, 3-8-1 Komaba, Meguro, Tokyo 153-8902, Japan

## Abstract

To understand the contribution of community effect on the stability of beating frequency in cardiac myocyte cell groups, the stepwise network formation of cells as the reconstructive approach using the on-chip agarose microchamber cell microcultivation system with photo-thermal etching method was applied. In the system, the shapes of agarose microstructures were changed step by step with photo-thermal etching of agarose-layer of the chip using a 1064-nm infrared focused laser beam to increase the interaction of cardiac myocyte cells during cultivation. First, individual rat cardiac myocyte in each microstructure were cultivated under isolated condition, and then connected them one by one through newly-created microchannels by photo-thermal etching to compare the contribution of community size for the magnitude of beating stability of the cell groups. Though the isolated individual cells have 50% fluctuation of beating frequency, their stability increased as the number of connected cells increased. And finally when the number reached to eight cells, they stabilized around the 10% fluctuation, which was the same magnitude of the tissue model cultivated on the dish. The result indicates the importance of the community size of cells to stabilize their performance for making cell-network model for using cells for monitoring their functions like the tissue model.

## Introduction

Development of reliable cell-based assay is important for high-speed, low cost drug screening. However, the conventional method using cells are still unstable and thus are still under trial to make reliable cell models showing the same extent of reliability as tissue/organ models. As heart is one of the most important organs for toxicology in drug screening, the properties of heart cells are examined and reported strenuously. For example, it has been reported that one beating cell can influence the rate of a neighbor with which it makes contact, and that a group of heart cells in culture, beating synchronously with a rapid rhythm, can act as pacemaker for a contiguous cell sheet from earlier tissue culture studies of cardiac myocyte cells [1]. Although these former results predicted that the importance of a rapidly beating region of tissue acts as pacemaker for a slower one and examined how the synchronization process of two isolated beating cardiac myocytes [2] and that the importance of the communication of each cells in the cell-network, the community size effect could not be measured successfully using the conventional cultivation method on the culture dish plate. As means of attaining the spatial arrangement of cardiac myocytes even during cultivation, we have developed a new single-cell based cultivation method and a system using agarose microstructures, based on 1064-nm photo-thermal etching [3-5]. Using this system, we measured the time course of synchronization process of adjacent two beating cardiac myocyte cells connected by 2-μm-width pathways, and found the synchronization of two cells occurred 90 min after their first physical contact [6,7].

This paper reports the cell network size effect (community effect) for stabilizing their beating intervals using our on-chip single-cell-based cultivation assay with stepwise modification of micorcultivation chamber structures during cultivation.

## Results

**Figure 1:**
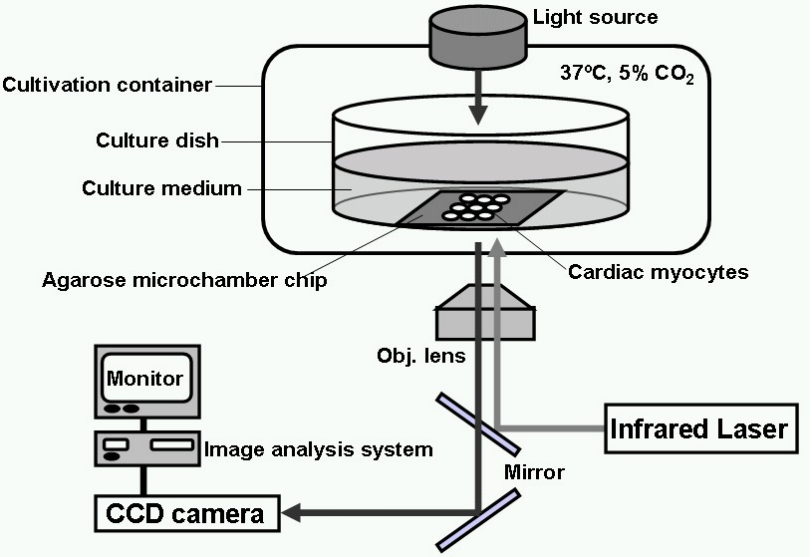
Schematic drawing of the on-chip single-cell-based cardiac myocyte network cultivation assay. A phase contrast microscope was used to measure the contraction rhythm of the cardiac myocytes and to melt a portion of agarose layer on the chip for the stepwise network formation of cells in the microchambers. The spontaneous beating rhythm of cultured cardiac myocytes is evaluated by the image analysis system, in which the change of the size (cross-section of volume) of each cardiac myocyte is analyzed and recorded every 1/30 s.

**Figure 2:**
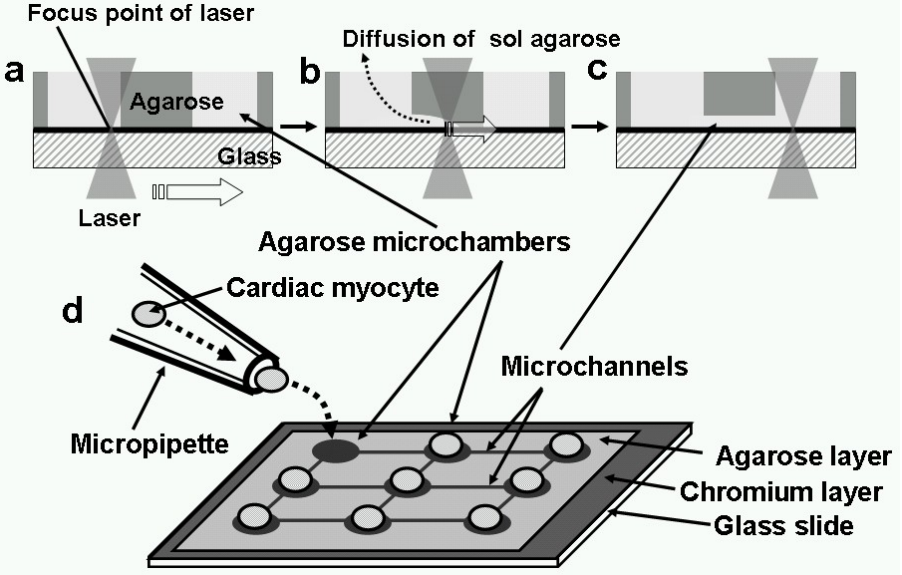
Schematic drawing of the photo-thermal etching method (a-c), and the design cell cultivation chip (d).

**Figure 3:**
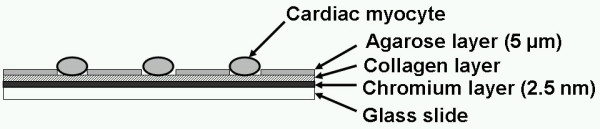
Cross-sectional view of the cell cultivation chip.

The schematic drawing of the on-chip single-cell-based cultivation assay is illustrated on Figure 1. Our system consists of three parts: temperature-controlled cell cultivation part, in which single cardiac myocytes are arranged in each microchambers of agarose cell cultivation chip; photo-thermal etching system to fabricate both the microchambers and microchannels by melting of a porting of the 5-μm-thick agarose layer by the spot heating of a 1064-nm infrared focused laser beam; and image acquire/analysis system. Figure 2 summarizes the chip design and cell cultivation procedure on the chip. In this example, we arranged nine 30-μm-diameter microchambers on the chip and the adjacent chambers are connected by the microchannels, which are fabricated during cultivation by the photo-thermal etching. As the 1064-nm laser beam is not absorbed by either water or agarose, it melts a portion of the agarose on the chromium thin layer because only the chromium layer absorbs the beam. Using this non-contact etching, we can easily make microstructures such as holes and channels within only a few minutes without using any cast molding process. The melting of agarose by laser occurred as follows (see Figure 2). We have focused the 1064-nm infrared laser beam on the agarose layer on the glass slide to melt the agarose at the focal point and on the light pathway until the shape of the hole for cells formed (Figure 2a). When the focused beam was moved parallel to the chip surface, a portion of agarose around the focal spot of laser melted and diffused into water (Figure 2b). After the heated spot had been moved, a channel was created at the bottom of the agarose layer connecting the two adjacent holes (Figure 2c). A microscope observation confirmed that the melting had occurred, and then either the heating was continued until the spot size reached the desired one, or the heating position was shifted to achieve the desired shape. Individual cardiac myocytes were cultivated in each hole of the agarose microchambers on the chip as shown in Figure 2d. In our method we added micro channels one by one to connect neighbouring cardiac myocytes in adjacent microchambers during their cultivation. To improve the attachment of the cells to the bottom of the microchambers, collagen-type I (Nitta gelatin, Osaka, Japan) was coated on the chromium layer of the chip before coating of agarose layer (Figure 3).

**Figure 4:**
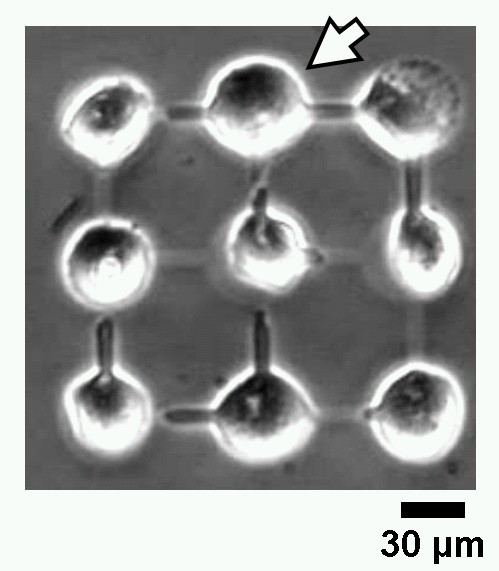
Optical micrograph of 24-h cultivation of nine cardiac myocyte cells' network.

A micrograph on Figure 4 shows the nine isolated cells cultivated in the nine-chamber agarose microcultivation chip (24 h following the beginning of the cultivation) and two isolated, independently beating cardiac myocytes coming into contact through the microchannel. During the cultivation, the beating frequency change of the cell indicated by the arrow was continuously observed.

**Figure 5:**
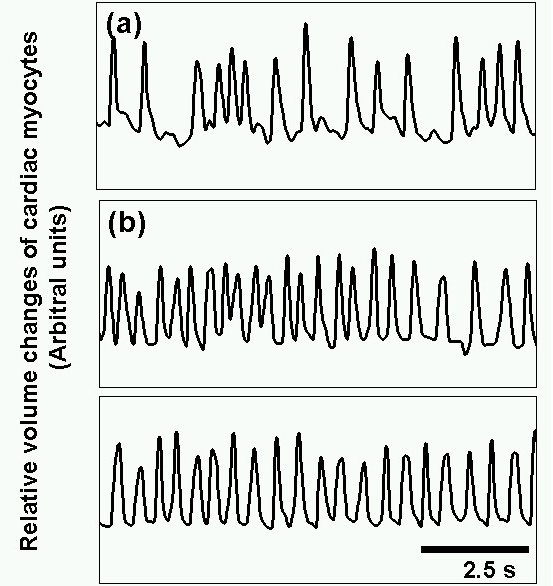
Time courses of cardiac myocytes of isolated single cell (a), two-cells network (b), nine-cells network (c), respectively.

**Figure 6:**
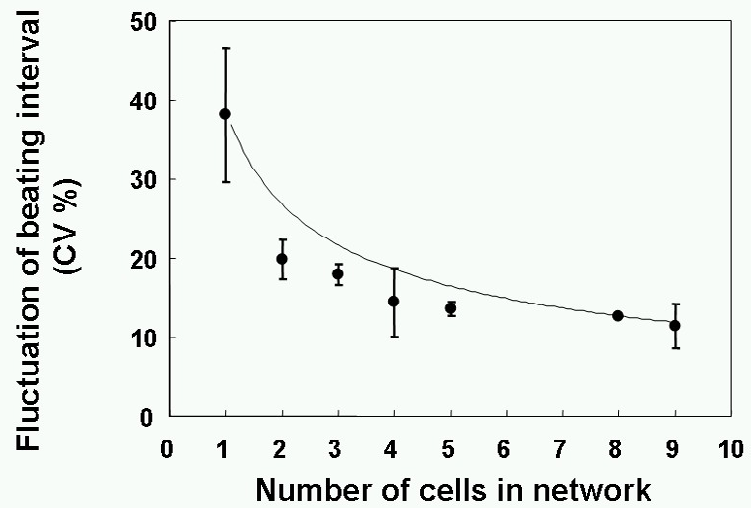
Cell network size dependence on the fluctuation of cardiac myocytes' beating frequency. In the graph, the mean values and standard deviations of nine sets of samples are indicated.

The time course change of the heart beating caused by the stepwise additional network formation was as shown in Figure 5. The frequency of isolated single cardiac myocyte cell's beating was fluctuated from the mean value (2.1 Hz, see Figure 5a). The beatings of the two-cell network (Figure 5b), in which one of the two cardiac myocytes was the same one as shown in Figure 5a. This two-cell network showed the much more stabilized beating than the isolated condition. We have further added more channels to the above two-cell network to form nine-cell network model. As shown in the graph in Figure 5c, the nine-cells network showed almost constant frequency of beating. The result of nine sets of different samples (one of the five sets was shown in Figure 5) summarized in Figure 6. As shown in the graph, the fluctuation of beating frequency was decreased according to the additional network formation of cardiac myocyte cells. In other words, the increase of network size improves the stability of the beating frequency. It should be noted that the magnitude of fluctuation for nine-cell network was about 10%, which was the same value of tissue culture sample of the same sample cultivated on the plate (data not shown). Moreover, we think we confirmed that the stepwise additional formation of channels during the cultivation by photo-thermal etching did not damage their beating ability just same as the stepwise formation of the neural network we have reported in previous papers [5,8].

The above results indicate two facts. First, for the single cell based research as the cell-network model, the consideration of community size of cell group is important for acquiring the reliable, stable data. Second, the community size required for reliable measurement like tissue model is not so large, i.e. nine cells were enough in this case. That might mean that smaller than we expected number of cells is required to produce the same community effect as in the tissue model. And therefore the potential for creating individual-cell-based cell-network model might be practical for reliable drug screening assay especially for human organ model. Moreover, it should be noted that we succeeded in the separating of two factors affecting the beating synchronization in this system, gap-junction connection and physical stretching. In other words, using this system we can measure the effect of gap-junction connections on synchronization clearly. If we could not remove the effect of physical stretching caused by the physical contact of neighbouring cells, we could hardly clarify the effect of chemicals to inhibit gap-junction connections. Because the cluster of cells still synchronized by their physical stretching even after gap-junction was inhibited (data not shown). From this viewpoint, our system might be most beneficial in drug screening.

In conclusion, we applied the 1064-nm photo-thermal etching method and made the on-chip agarose single cell microcultivation system for generating cardiac myocyte networks of different size, which is important for understanding the community effect of rhythm synchronization. Using the system, we for the first time observed the differences in the synchronization process of cardiac myocyte cells and their dependence on the community size. This system can potentially be used in the biological/medical fields for cultivating next generation of networks from individual cultured cells and measuring their properties.

## Materials and Methods

Ventricular myocytes were isolated from 1- to 3-day-old neonatal Wistar rats as described earlier [6,7]. Hearts were excised from rats anaesthetized with ethyl ether and transferred to phosphate buffered saline (PBS, 137 mM NaCl, 2.7 mM KCl, 8 mM Na2HPO4, 1.5 mM KH2PO4, pH 7.4) containing 0.9 mM CaCl2 and 0.5 mM MgCl2. after which ventricles were separated and minced into small fragments. Tissue fragments were further dissociated by incubating them twice with PBS containing 0.25% collagenase (Wako, Osaka, Japan) for 30 minutes at 37°C. The cell suspensions were transferred to a cell culture medium (DMEM [Invitrogen Corp., Carlsbad, CA USA] supplemented with 10% fetal bovine serum, 100 U/ml penicillin, and 100 μg/ml Streptomycin) at 4°C. The cells were filtered through a 40-μm nylon mesh and were centrifuged at 180 g for 5 minutes at room temperature. The cell pellet was re-suspended in a HEPES buffer (20 mM HEPES, 110 mM NaCl, 1 mM NaH_2_PO_4_, 5 mM glucose, 5 mM KCl, and 1 mM MgSO_4_, pH 7.4). The cardiac myocytes present in the suspension were separated from other cells (i.e., fibroblasts and endothelial cells) by the density centrifugation method. The cell suspension was then layered onto 40.5% Percoll (Amersham Biosciences, Uppsala, Sweden) diluted in the HEPES buffer, which had previously been layered on 58.5% Percoll diluted in the buffer. The cell suspension was then centrifuged at 2200 g for 30 minutes at room temperature. Cardiac myocytes were retrieved from the interface of the 40.5% and 58.5% Percoll concentrations. Retrieved cells were then re-suspended in the cell culture medium. The 5-μl of the suspension, which was diluted to achieve a final concentration of 3.0 × 10^5 ^cells/ml, was plated into the chip and each cardiac myocyte was picked up by a micropipette and manually introduced into each microchamber in the chip. Then, it was incubated on a cell-cultivation microscope system at 37°C in a humidified atmosphere of 95% air and 5% CO_2_. It should be noted that, because the microchamber sidewalls were made of agarose, the cells could not easily pass over the chambers. A phase-contrast microscope was used both to measure the contraction rhythm (i.e. beating frequency) of the cardiac myocytes, and to record the shape of cell network in microchambers.

The spontaneous contraction rhythm of cultured cardiac myocytes was evaluated by a video-image recording method. Images of beating cardiac myocytes were recorded with a CCD camera through the use of a phase contrast microscope. The sizes (cross-sectional area of cell) of cardiac myocytes, which changed considerably with contraction, were also analyzed and recorded every 1/30 s by a personal computer with a video capture board and estimated their beating phenomenon by the change of their cross-sectional area sizes [6,7].

## Authors' contributions

KK and TK carried out the microchamber design, cell preparation, single cell cultivation and observation, image analysis. They were equally contributed for this article. KY conceived of the study, and participated in its design and coordination. All authors read and approved the final manuscript.
